# A Standardized Telephone Intervention Algorithm Improves the Survival of Ventricular Assist Device Outpatients

**DOI:** 10.1111/aor.13155

**Published:** 2018-05-25

**Authors:** Thomas Schlöglhofer, Johann Horvat, Francesco Moscato, Zeno Hartner, Georg Necid, Harald Schwingenschlögl, Julia Riebandt, Kamen Dimitrov, Philipp Angleitner, Dominik Wiedemann, Günther Laufer, Daniel Zimpfer, Heinrich Schima

**Affiliations:** ^1^ Center for Medical Physics and Biomedical Engineering Medical University of Vienna Vienna Austria; ^2^ Department of Cardiac Surgery Medical University of Vienna Vienna Austria; ^3^ Ludwig‐Boltzmann‐Cluster for Cardiovascular Research Vienna Austria

**Keywords:** Ventricular assist device, —Mechanical circulatory support, —Outpatient management, —Readmission, —Algorithm

## Abstract

Ventricular assist devices (VADs) are an established therapeutic option for patients with chronic heart failure. Continuous monitoring of VAD parameters and their adherence to guidelines are crucial to detect problems in an early stage to optimize outcomes. A telephone intervention algorithm for VAD outpatients was developed, clinically implemented and evaluated. During the phone calls, a structured inquiry of pump parameters, alarms, blood pressure, INR, body weight and temperature, exit‐site status and heart failure symptoms was performed and electronically categorized by an algorithm into 5 levels of severity. VAD outpatient outcomes without (*n* = 71) and with bi‐weekly telephone interviews in their usual care (*n* = 25) were conducted using proportional hazard Cox regression, with risk adjustment based on a propensity score model computed from demographics and risk factors. From February 2015 through October 2017, 25 patients (*n* = 3 HeartMate II, *n* = 4 HeartMate 3 and *n* = 18 HeartWare HVAD) underwent 637 telephone interventions. In 57.5% of the calls no problems were identified, 3.9% were recalled on the next day because of alarms. In 26.5% (*n* = 169), the VAD Coordinator had to refer to the physician due to elevated blood pressure (*n* = 125, >85 mm Hg), INR < 2.0 or > 4.0 (*n* = 24) or edema (*n* = 10), 11.9% of the calls led to a follow‐up because of equipment or exit‐site problems. Propensity‐adjusted 2‐year survival (89% vs. 57%, *P* = 0.027) was significantly higher for the telephone intervention group. Continuous, standardized communication with VAD outpatients is important for early detection of upcoming problems and leads to significantly improved survival.

Ventricular assist devices (VADs) are an established therapeutic option for patients with end‐stage heart failure 1. Continuous monitoring of pump parameters and adherence to patient management guidelines 2 are crucial to detect problems at an early stage to reduce the number of adverse events and to optimize outcomes. Recent studies 3, 4 have demonstrated that sub‐therapeutic international normalized ratio (INR) and elevated mean arterial blood pressure (MAP) exceeding 90 mm Hg are independent risk factors for pump thrombosis and strokes. With commonly available rotary blood pumps (RBPs), only 30% of patients are free from any first infection, bleeding or stroke after 12 months of support 5. Some of these adverse events and readmissions can possibly be prevented by regular assessment and optimization of pump parameters, strict hypertension management, and mandatory clinical follow‐ups 6. Especially variable from center to center is the frequency of outpatient follow up visits, which depends on the number of ongoing VAD patients and available resources, patient condition and the distance to the implanting center.

With the establishment of destination therapy (DT) in patients with advanced heart failure, VAD clinicians are faced with the aspect of longer support duration extending into years. While the application of mechanical circulatory support (MCS) as DT has evolved, international experts remain uncertain about the intensity of follow‐up care needed to achieve optimal outcomes 7. In spite of evidence that enhanced surveillance of outpatients adds to the quality of care. Intervention programs based on comprehensive care and intensive follow‐up by a multidisciplinary team have achieved promising reduction in mortality in chronic heart failure 8 as well as VAD patients 7.

To further this reduction, a telephone intervention algorithm for constant communication with VAD outpatients was developed, clinically implemented, and evaluated. The particular aim of this single center study was to test the hypothesis that telephone interventions of left ventricular assist device (LVAD) patients, performed by VAD Coordinators, could aid early identification of potential adverse events and allow earlier interventions leading to a significant reduction in mortality when compared to usual care.

## Patients and Methods

**Figure 1:**
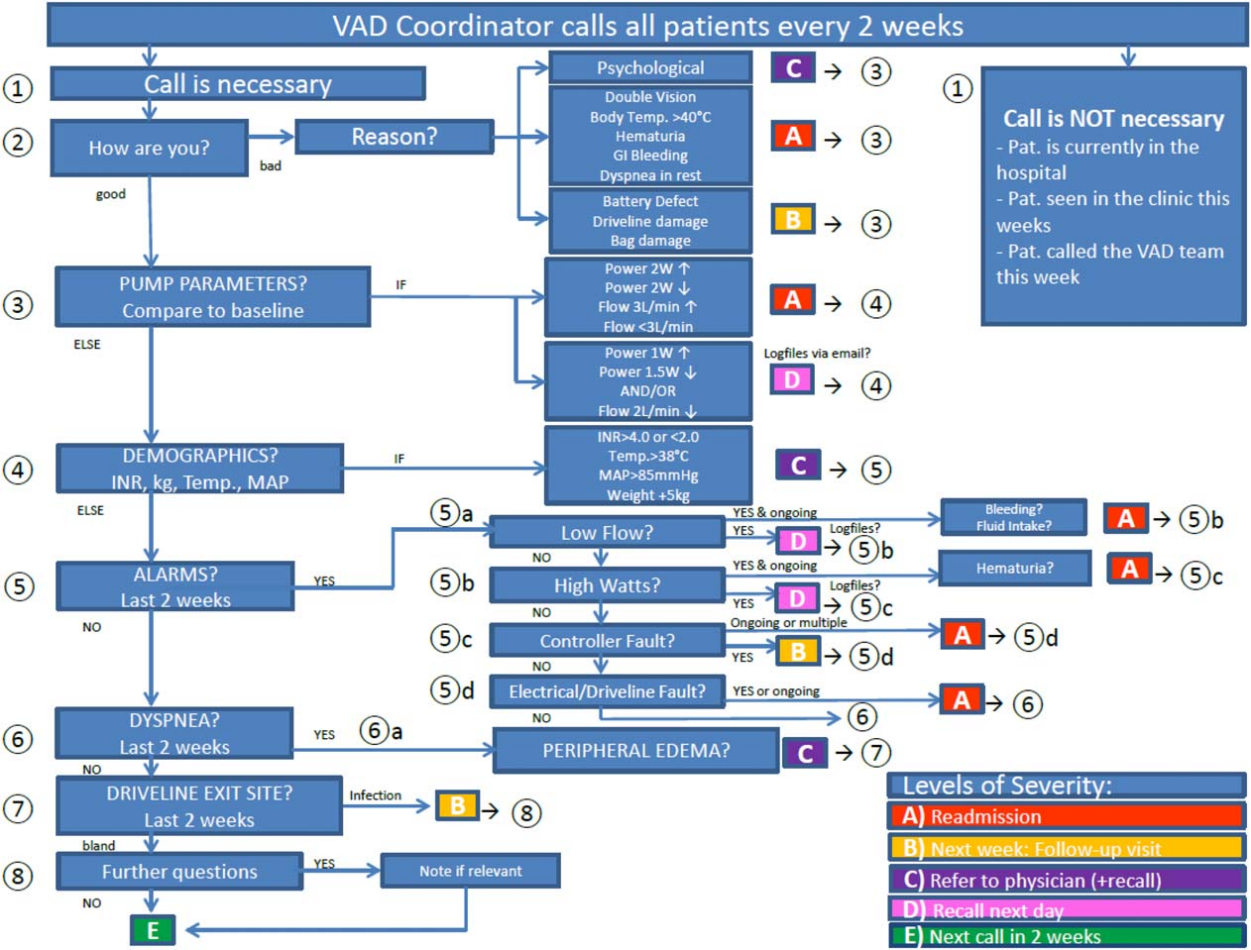
Flow chart of the telephone intervention algorithm. Five levels of severity: Readmission (A), follow up visit in the outpatient department in the next week (B), refer problem to the physician including possible recall of the patient by the VAD Coordinator (C), Recall by the VAD Coordinator on the next day (D), No problems detected – next call in 2 weeks (E). INR, international normalized ratio; kg, weight; Temp, body temperature; MAP, mean arterial pressure; VAD, ventricular assist device; GI, gastrointestinal. [Color figure can be viewed at http://wileyonlinelibrary.com]

The study compared usual care (control group) to VAD outpatients who received bi‐weekly standardized telephone interventions (Fig. 1) additional to their usual care (intervention group). The survey questions were developed by consensus of the VAD team based on a previous study 9, that assessed the quality of anticoagulation and antihypertensive therapy and evaluated pump parameters and possible symptoms of heart failure. The study was approved by the institutional review board. Since January 2015, all VAD outpatients gave informed consent and were then assigned to the telephone intervention group. The first *n* = 25 patients implanted after the clinical telephone intervention implementation were assigned to the study cohort. Freedom to decline and anonymity in all reported data were guaranteed.

### Telephone intervention algorithm

Subjects in the usual care (UC) group continued treatment with the VAD team in the same manner as in the telephone intervention (TI) group, except for bi‐weekly phone calls. In both groups, frequency of outpatient clinic visits (at intervals of 2 to 3 months) and medical therapy postdischarge was comparable, including phenprocoumon anticoagulation therapy (international normalized ratio (INR) target range: 2.0–3.0), acetylsalicylic acid (ASA) antiplatelet monotherapy (daily dose: 100–200 mg), and MAP target < 90 mm Hg. The adherence to the INR target range was assessed by the percent of time in therapeutic range (TTR) based on the Rosendaal method 10.

**Figure 2:**
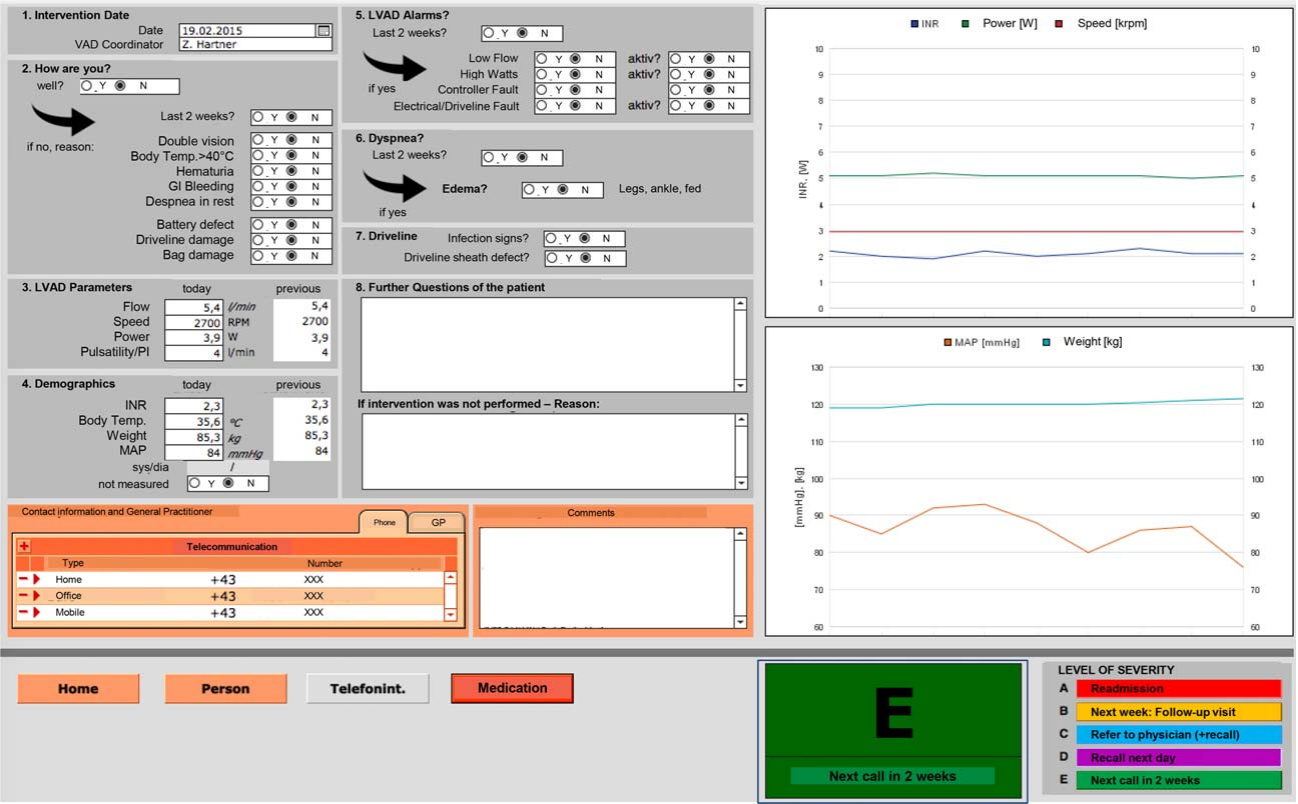
Graphical user interface of the telephone intervention algorithm. [Color figure can be viewed at http://wileyonlinelibrary.com]

Patients assigned to the TI group were followed with a telephone intervention by a VAD Coordinator. No calls were placed if the patient was either currently admitted into hospital, or was seen in the clinic that week, or had already phone contact with the VAD team that week. During the telephone interventions, patients were initially asked an open‐ended question, followed by a structured inquiry about VAD parameters (speed, flow, power, and pulsatility/pulse index), alarms, MAP, INR, body weight and temperature, driveline exit‐site status, and symptoms of dyspnea and/or edema. The content of the intervention was documented electronically with a graphical user interface (Fig. 2) in the VAD database (Filemaker Pro 11, Filemaker Inc. Santa Clara, CA, USA) of our institute. Answers are automatically segregated, based on a self‐developed algorithm (Fig. 1), into one of five levels of severity and are triaged by the VAD Coordinator. Severities were classified into: immediate hospital readmission (A), follow‐up in the outpatient department in the next week (B), reference of the problem to the physician, including possible recall of the patient by the VAD Coordinator (C), recall on the next day (D), or—if no problem was detected—schedule for the next call in 2 weeks (E).

### Study design

**Figure 3:**
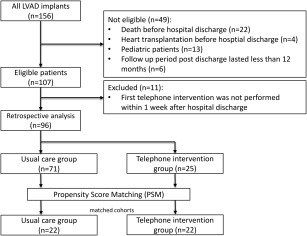
Flowchart of enrollment and exclusion of retrospectively analysis of patients who had undergone LVAD implantation at the Medical University of Vienna from January 2012 through November 2015.

This retrospective, single center study included 156 patients who received LVAD implantation between January 2012 and November 2015. Of these patients, 22 were not eligible because they did not survive to hospital discharge, received heart transplantation before hospital discharge (*n* = 4), were pediatric patients (*n* = 13), follow‐up was performed in another center (*n* = 4) or for patients recently placed on device, their follow‐up period post discharge lasted less than 12 months (*n* = 6). This triage resulted in 107 eligible patients (71 UC and 36 TI group) from which 11 patients were excluded because the first telephone intervention was not performed within 1 week following hospital discharge (Fig. 3).

### Study population

Ninety‐six patients who received a durable, long‐term VAD (HeartMate II and 3, Abbott Inc., Abbott Park, IL, USA; HVAD, Medtronic Inc., Minneapolis, MN, USA) as Bridge to Transplant (BTT), DT or Bridge to Candidacy (BTC) were included in the study. The maximum follow‐up for each patient was 24 months post discharge.

### Propensity score model

The UC and TI group showed differences in their demographics, risk factors and operative variables (Table 1). To minimize confounding the effects of the standardized telephone interventions on long term survival and readmission rates of VAD outpatients, propensity score matching (PSM) was used 11, 12, considering telephone intervention as treatment. To match the two groups of patients, a propensity score was derived from a nonparsimonious logistic multivariate model applied to all includable outpatients (*n* = 71 UC and *n* = 25 TI). Demographics and risk factors (only one variable was picked from among a closely related cluster of variables to represent the cluster) were included in the model, using the variables and categories described in Table 1. Additionally, a time‐related variable (day of initial hospital discharge) was included in the propensity model to account for possible differences in the patient health status and recovery from implantation procedure that could affect outpatient survival.

**Table 1:** Comparison of demographic, risk factor and operative data for the telephone intervention and routine care cohorts (unmatched and propensity score matched)

Variable/Category	Unmatched Cohorts			Propensity Score Matched Cohorts		
	Usual Care	Telephone Intervention	*P* value	Usual Care	Telephone Intervention	*P* value
Number of patients	*n* = 71	*n* = 25		*n* = 22	*n* = 22	
Demographics						
Male sex	87.3%	84.0%	0.68	86.4%	81.8%	0.69
Age (years)	59.8 ± 10.5	56.8 ± 12.6	0.36	59.3 ± 13.3	59.9 ± 9.3	0.86
Body mass index (kg/m^2^)	26.5 ± 4.6	26.2 ± 5.8	0.41	25.3 ± 3.8	26.3 ± 6.2	0.83
Underlying disease			0.52			0.76
Ischemic cardiomyopathy	63.4%	56.0%		54.5%	59.1%	
Dilated cardiomyopathy	36.6%	44.0%		45.5%	40.9%	
Intermacs Level 1, 2, 3, 4, 5, 6, 7	2.6 ± 1.2	2.9 ± 1.5	0.34	2.8 ± 0.8	2.9 ± 1.1	0.65
Indication			0.57			0.67
Bridge to transplant	29.6%	32.0%		36.4%	31.8%	
Bridge to candidacy	35.2%	40.0%		27.2%	36.4%	
Destination therapy	35.2%	28.0%		36.4%	31.8%	
Surgical access			0.05			0.75
Sternotomy	23.9%	44.0%		31.8%	36.4%	
Minimal invasive	76.1%	56.0%		68.2%	63.6%	
Devices			0.77			0.99
Medtronic HVAD	62.0%	72.0%		68.2%	72.7%	
Abbott HeartMate II	38.0%	12.0%		31.8%	13.6%	
Abbott HeartMate 3	0.0%	16.0%		0.0%	13.6%	
Initial hospital discharge (days)	52.8 ± 30.3	64.6 ± 58.9	0.35	58.0 ± 36.6	56.1 ± 51.3	0.84

Data presented as % or mean ± SD.

This propensity score was used in four complementary analyses: propensity–adjusted TI (*n* = 22) versus UC (*n* = 22) group comparison of (i) 2‐year post discharge survival, (ii) 2‐year post discharge freedom from any readmission, (iii) reasons for readmission including total length of hospital stay following 2 years after discharge, and (iv) mean arterial blood pressure (MAP) during regular outpatient follow‐up.

### Statistical analysis

Statistical Analysis was performed using SPSS for Windows Release 23.0.0 (SPSS Inc., Chicago, IL, USA) and R statistical software for Windows, version 3.3.0 (R Development Core Team, Auckland, New Zealand). Characteristics of patients with and without telephone intervention are reported as mean ± SD, compared with the Fisher exact test for categorical and the Student's *t*‐test (normally distributed) or Mann‐Whitney *U* test (non‐normally distributed) for continuous variables, respectively. Normal distribution was assessed by the Shapiro‐Wilk test. Propensity‐adjusted survival and freedom from any readmission was analyzed using Kaplan‐Meier curves and compared with the log‐rank test. In all analyses, a value of *P* < 0.05 was considered as statistically significant.

## Results

### Baseline characteristics of the patients

In total, *n* = 96 outpatients who had undergone LVAD implantation between January 2012 and November 2015 were retrospectively analyzed. Of these, *n* = 71 received UC and *n* = 25 bi‐weekly telephone interventions additionally to their UC. Baseline characteristics of the two groups were significantly different. Therefore, a propensity score was derived and applied to all includable VAD outpatients to match the two groups of patients. This PSM resulted in a risk adjusted matched dataset of *n* = 22 UC and *n* = 22 TI patients (Table 1) with no significant differences in their comorbidity, preoperative diagnostic data or postoperative anticoagulation and antiplatelet therapy (Table 2).

**Table 2:** Comorbidity, preoperative diagnostic data, postoperative anticoagulation target range and daily antiplatelet dose in propensity matched usual care (n = 22) and telephone intervention (n = 22) group

Variable/Category	Comorbidity, preoperative		
	Usual Care	Telephone Intervention	*P* value
Number of patients	*n* = 22	*n* = 22	
Preoperative comorbidity data			
Heart attack, present	18.2%	31.8%	0.30
Coronary heart disease, present	36.4%	50.0%	0.37
Diabetes, present	13.6%	31.8%	0.16
Pulmonary hypertension, present	18.2%	18.2%	1.00
Arterial hypertension, present	18.2%	27.3%	0.48
ECMO, present	9.1%	9.1%	>0.99
Preoperative laboratory parameters			
Creatinine (mg/dL)	1.52 ± 0.69	1.36 ± 0.45	0.77
Leukocytes (G/L)	8.24 ± 3.54	8.96 ± 3.33	0.15
Gamma GT (U/L)	132.5 ± 130.2	178.5 ± 228.9	0.66
Blood urea nitrogen (mg/dL)	30.22 ± 16.93	27.68 ± 12.07	0.69
Total bilirubin (mg/dL)	1.48 ± 0.49	1.17 ± 0.65	0.054
Fibrinogen (mg/dL)	403.5 ± 120.7	475.7 ± 153.9	0.062
Albumin (g/L)	35.45 ± 6.34	34.12 ± 6.38	0.47
Postoperative medical therapy			
INR range:			0.30
2.0–2.3	13.6%	4.5%	
2.0–2.5	81.8%	81.8%	
2.5–3.0	4.5%	13.6%	
ASA daily dose (mg)	122.7 ± 61.2	147.7 ± 69.8	0.25

Data presented as % or mean ± SD.

ECMO, extracorporeal membrane oxygenation; ASA, acetylsalicylic acid; INR, international normalized ratio.

Of the 44 patients included in the risk adjusted cohort, 15 (34%) received a VAD as BTT, 15 (34%) as DT and 14 (32%) as BTC. Mean age of the patients was 59.6 ± 11.34 years (range 19–77 years), and 16% were female. The cause of cardiomyopathy was dilated in 19 patients (43%). Three different devices (*n* = 31 HeartWare HVAD, *n* = 10 HeartMate II and *n* = 3 HeartMate 3) were implanted. Surgical access was a full sternotomy (34%) or bilateral mini‐thoracotomy (66%). Following discharge, postoperative medical therapy included oral phenprocoumon (vitamin K‐antagonist) anticoagulation therapy, with an INR target range of 2.0–2.5 in 82% of the patients and acetylsalicylic acid (ASA) antiplatelet monotherapy with an average daily dose of 135.2 ± 66.1 mg.

### Analysis of telephone interventions

**Figure 4:**
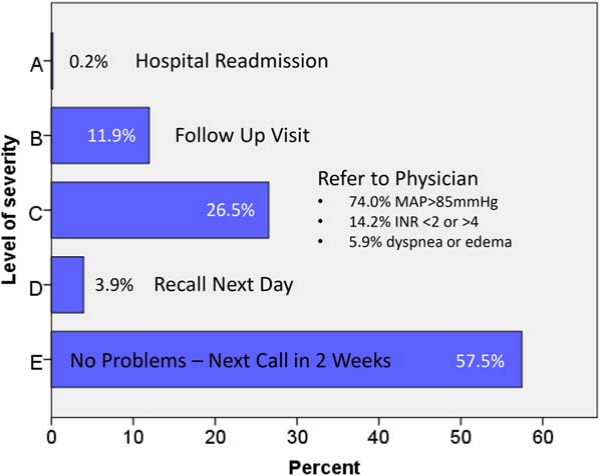
Results of the severity classification of *n* = 637 telephone interventions of 25 patients of the telephone intervention group (*n* = 3 Abbott HeartMate II and *n* = 4 HeartMate 3, *n* = 18 Medtronic HVAD). INR, international normalized ratio; MAP, mean arterial pressure. [Color figure can be viewed at http://wileyonlinelibrary.com]

From February 2015 through October 2017, the 25 patients of the TI group underwent 637 telephone interventions (Fig. 4). Within 2 years after initial discharge, 57.5% (*n* = 366) of the calls revealed no problems as patients adhered well to institutional guidelines of blood pressure, INR, and pump parameters were within normal operation. Thus, all calls resulted in scheduling the next call in 2 weeks. In 3.9% (*n* = 25) of the cases, patients were recalled by the VAD Coordinator on the next day to reassess the patient status because of alarms (e.g., low flow) or minor changes in pump parameters. In 26.5% (*n* = 169), the VAD Coordinator had to refer to the physician due to elevated MAP or body temperature > 38°C, INR out of range, or a body weight increase of more than +5 kg over the preceding 2 weeks. The most common reasons for assigning the 26.5% calls entering category C were a MAP > 85 mm Hg (*n* = 125), INR <2.0 or >4.0 (*n* = 24), or new symptoms of heart failure, for example, peripheral edema and/or dyspnea (*n* = 10). Overall, 11.9% (*n* = 76) of the telephone interventions resulted in a follow‐up visit in the following week because of peripheral device problems (e.g., defective battery or patient bag, outer sheath driveline damage) or driveline exit‐site infections. Only 0.2% (*n* = 1) of the calls were assigned to category A and resulted in an immediate readmission. Most of the calls (95.6%) lead to a single severity classification and 4.4% (*n* = 28) to multiple categories (e.g., B + C ‐ driveline infection in combination with elevated MAP). 42.0% (*n* = 110) of the detected and classified problems were emerging problems, whereas 58.0% (*n* = 152) were problems that were already known or under treatment, such as a INR < 2.0 in patients on low‐molecular‐weight heparin (Enoxaparin) therapy, driveline infection under medical treatment, or elevated MAP > 85 mm Hg consequent to elevated antihypertensive therapy by the VAD team within the last week.

### Effect of the telephone intervention

Telephone intervention was associated with a significantly reduced MAP measured during regular outpatient follow‐up in the hospital by the VAD Coordinator: 86.3 ± 12.4 mm Hg (UC) versus 82.8 ± 9.3 mm Hg (TI), *P* = 0.02. Within 24 months post discharge, the frequency of elevated MAP (>90 mm Hg) during regular follow‐up was significant lower (*P* = 0.047) in TI patients: 22.7% (UC) versus 31.8% (TI) had no, 9.1% (UC) versus 31.8% (TI) one, 36.4% (UC) versus 27.3% (TI) two, and 31.8% (UC) versus 9.1% (TI) multiple (≥3) times elevated MAP during regular follow‐up.

Patients in the TI group adhered well to the institutional anticoagulation therapy guidelines. Patient self‐testing of INR was performed with the CoaguCheck XS (Roche, Roche Diagnostics, Mannheim, Germany). Of *n* = 622 assessed INR measurements in the TI group, INR was 2.4 ± 0.4 with a mean time in therapeutic range (TTR) of 72.2 ± 13.6% and 72.4 ± 13.0% of tests in the target range.

### Readmission

**Figure 5:**
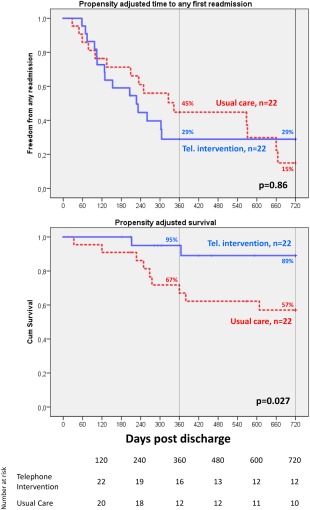
Comparison of risk‐adjusted freedom from any readmission (top) and late survival (bottom) 2 years post initial discharge for the telephone intervention versus usual care (no telephone intervention) group. Risk adjustment was done with a telephone intervention use propensity score adjustment. [Color figure can be viewed at http://wileyonlinelibrary.com]

Within 2 years following initial discharge, 32% UC and 26% TI patients had one readmission, 53% UC and 45% TI patients had multiple readmissions (≥2), with no significant difference of the average time to any first readmission (321.3 ± 244.9 days versus 298.3 ± 246.1 days, *P* = 0.89, Fig. 5 top). Patients were readmitted a total of 74 times, with a cumulative rate of 1.55 ± 1.5 (UC) versus 1.82 ± 1.9 (TI) times/patient, as of the end of follow‐up.

Freedom from any readmission (15% vs. 29%, *P* = 0.86, Fig. 5 top) as well the average days of rehospitalization in the hospital (32.9 ± 46.4 vs. 18.4 ± 22.7 days, *P* = 0.64) within 2‐years post discharge was nonsignificantly higher in the UC versus TI group. Patients were supported for a total of 23 173 days of which 89.5% (UC) versus 96.3% (TI), *P* = 0.60, were spent out of hospital.

At 24 months post discharge, the most common etiology for readmission was major bleeding (including gastrointestinal bleeding) in 36.4% of the UC (vs. 20.8% TI, *P* = 0.20) and other causes (e.g., dizziness, malnutrition, altered mental status, abnormal blood glucose, fall or social indication) in 45.8% of the TI patients (vs. 22.7% UC, *P* = 0.09). Neurological dysfunction was comparable between groups (UC 27.3% vs. TI 20.8%, *P* = 0.43), although no readmission due to hemorrhagic cerebral strokes occurred in the TI group (vs. 16.1% of all neurological UC readmissions), representing the second most frequent clinical readmission profile in the UC group. 33.3% device‐related driveline or pocket infections as the primary cause of readmission represented the second most frequent reason in the TI cohort and occurred significantly more often than in the UC group (33.3% vs. 4.5% UC, *P* = 0.02). Pump thrombus related admissions were comparable between the groups (18.2% vs. 12.5% TI, *P* = 0.45), where all thrombus events could be successfully treated by thrombolytic therapy in the TI group, but pump exchange was necessary in 50% of the UC group.

### Survival

Kaplan‐Meier curves for the UC and TI group 2‐years post initial hospital discharge, are presented in Fig. 5. Risk‐adjusted survival (89% vs. 57% UC, *P* = 0.027) was significantly higher in the telephone intervention group.

Of those patients who expired during the follow up period, in the UC cohort nonsignificant more patients died at home than in the hospital (44.6% vs. 0.0% TI group, *P* = 0.44). Causes of death in the UC group included neurological dysfunction (*n* = 3), major infection (*n* = 3), major bleeding (*n* = 1), renal dysfunction (*n* = 1), and respiratory failure (*n* = 1). Only two patients in the TI group died after rehospitalization due to a neurological event (ischemic cerebral accident) on and a major infection on postdischarge day 212 and 365, respectively.

## Discussion

Although continuous flow blood pumps show good clinical outcomes and long‐term survival in BTT and DT patients 1, unplanned readmissions 6, 13, 14 are common following LVAD implantation and account for a significant percentage of healthcare costs 15 in this patient population. Gastrointestinal (GI) bleeding, strokes and driveline infections are the leading causes for these admissions. Preventing these adverse events or at least detecting them at an earlier, less urgent, stage must be the focus in VAD patient management to improve outcomes. While some centers report routine phone calls 7 or at least phone contact 16 to the patient or caregiver in addition to consensus‐based VAD outpatient practices, this is the first study reporting the implementation of a standardized telephone intervention algorithm. By means of a graphical user interface all problems detected on call were categorized fully, automatically based on an algorithm in five levels of severity and triaged in a standardized manner by the VAD Coordinator.

In the analysis presented here, we attempted to address the effect of confounding factors that might have influenced the observed significant improved survival of the TI versus UC group by a PSM analysis. A possible contributing factor to improved survival attributable to a learning curve in improved patient selection and perioperative management can be excluded as the VAD implant volume in our center was stable over the entire study period and all patients were treated in the same manner with the same medical therapy postdischarge.

We suggest that enhanced monitoring based on telephone interventions contributes to the earlier detection and even avoidance of severe adverse events. This is evidenced first at 24 months post hospital discharge where we observed a significantly better survival in the TI outpatient cohort without any out‐of‐hospital death, whereas 44.6% of the UC patients died at home, where their problems were either not detected or were detected too late. A second potential benefit was a nonsignificantly higher freedom from any readmission in the TI group (15% vs. 29% UC), especially when considering the readmission clinical profiles at 24 months post discharge, it becomes evident that TI might lead to earlier adverse event detection. Although pump thrombus related admissions were comparable between the groups (12.5% vs. 18.2% UC, *P* = 0.45), all of them could be successfully treated by medical therapy in the TI group, but 50% of UC patients required a pump exchange as the pump thrombus was already manifested. Driveline infection as the primary cause of readmission occurred significantly more often in the TI group (33.3% vs. 4.5% UC, *P* = 0.02). Whereas UC and TI patients have the same potential risk for driveline infections, they were potentially undiagnosed in UC patients as they were unlikely to communicate their wound status to the VAD center if not actively asked for it. Thus, telephone intervention might lead to earlier detection of driveline infections allowing adequate treatment of them during an additional follow up in the next week (category B), in possible comparison to *n* = 3 UC patients who died at home due to sepsis following severe driveline infection. Other readmission causes which can be treated faster and easier, at less expense 15, accounted for 22.7% in the UC and 45.8% in the TI group. We note the cumulative shorter length of hospital stay for readmissions over 2‐years post discharge in the TI group of 18.4 days versus 32.9 days in the UC group, pointing to greater number of readmissions based on other, “less urgent” causes in the TI cohort. Although the number of TI and UC patients showing freedom from any readmission after 2 years is comparable, telephone interventions may improve cost effectiveness of VAD therapy because they could cut direct hospital costs by reducing admitted days in the hospital.

Another possible contribution to improved survival in the TI group is better adherence to institutional guidelines for anticoagulation and hypertension management: Previous studies revealed that VAD patients spent only 50–60% of their TTR 17, 18, 19 and only 11.4% of patients transmit their INR readings telemetrically to the VAD center 20, suggesting room for improvement. In this study, patients in the TI cohort reported a mean INR of 2.4 ± 0.4 and spent 72.2% of the TTR, possibly contributing to the reduced readmission rates based on major bleeding of 20.8% (TI) versus 36.4% in the UC group. This interpretation is supported by previous reports 16, 21 that bleeding is the most common postoperative event in these patients. As VAD patients with elevated MAP are at an increased risk for pump thrombus or stroke 3, 4, 22, another positive effect of the telephone intervention algorithm is the enhanced blood pressure management seen in the TI versus UC group, resulting in a significantly lower MAP during follow up. Additionally, within 24 months post discharge, the prevalence of MAP measurements >90 mm Hg was significantly reduced in TI versus UC patients (e.g., ≥3 times: 9.1% vs. 31.8% UC, *P* = 0.047).

Finally, psychological factors may contribute to the better outcomes of TI patients. The routinely performed query of pump parameters during the telephone intervention could raise the patients' desire to know their normals (e.g., power consumption, weight, etc.) and to communicate abnormalities to the VAD team immediately. Based on these regular calls, the inhibition level of patients to call the 24/7 emergency hotline in case of alarms or problems may be lower, thus leading to more frequent readmissions but earlier detection of adverse events. Patient acceptance of this new treatment tool was high, as only one of 25 patients refused the frequent intervention after 11 months, but asked for resumption in the program after 2 months of suspension. As frequent follow‐up schedules are difficult to sustain with a large number of VAD‐supported patients potentially spread over a larger geographical area, this novel tool for VAD outpatient management may well be a useful additional approach to usual care.

This retrospective study was performed at a single center; therefore, generalizability may be limited. The algorithm was applied nonrandomized to all VAD outpatients as this enhanced patient management strategy evolved as “best practices” in our center. Additionally, the rather small sample size limits interpretation and statistical power. A larger multicenter study might better characterize VAD outpatient management with and without telephone intervention than was possible in this study.

## Conclusions

Continuous, standardized telephone interventions with ventricular assist device outpatients enable a constant communication and fast information transfer, which allows an early detection of upcoming problems with an immediate benefit for survival and severity reduction of adverse events compared to usual oversight of VAD outpatients.

## Author Contributions

TS, DZ and HS developed the concept and design, TS and FM performed the statistical analysis, funding was secured by DZ, GL and HS, TS drafted the article, TS, JH, ZH, GN, HS, JR, KD, PA and DW collected the data.

## Conflict of Interest

None of the authors has any financial relationship related to this manuscript to disclose.
